# Gut microbiota and cardiovascular disease: opportunities and challenges

**DOI:** 10.1186/s40168-020-00821-0

**Published:** 2020-03-14

**Authors:** Negin Kazemian, Morteza Mahmoudi, Frank Halperin, Joseph C. Wu, Sepideh Pakpour

**Affiliations:** 1 School of Engineering, University of British Columbia, Kelowna, Kelowna, BC Canada; 2 Department of Radiology and Precision Health Program, Michigan State University, East Lansing, MI USA; 3 Cardiology Interior Health, Kelowna, BC Canada; 4 Stanford Cardiovascular Institute, Stanford University School of Medicine, Stanford, CA USA; 5 Department of Medicine, Stanford University School of Medicine, Stanford, CA USA; 6 Institute for Stem Cell Biology and Regenerative Medicine, Stanford University School of Medicine, Stanford, CA USA

## Abstract

Coronary artery disease (CAD) is the most common health problem worldwide and remains the leading cause of morbidity and mortality. Over the past decade, it has become clear that the inhabitants of our gut, the gut microbiota, play a vital role in human metabolism, immunity, and reactions to diseases, including CAD. Although correlations have been shown between CAD and the gut microbiota, demonstration of potential causal relationships is much more complex and challenging. In this review, we will discuss the potential direct and indirect causal roots between gut microbiota and CAD development via microbial metabolites and interaction with the immune system. Uncovering the causal relationship of gut microbiota and CAD development can lead to novel microbiome-based preventative and therapeutic interventions. However, an interdisciplinary approach is required to shed light on gut bacterial-mediated mechanisms (e.g., using advanced nanomedicine technologies and incorporation of demographic factors such as age, sex, and ethnicity) to enable efficacious and high-precision preventative and therapeutic strategies for CAD.

## Key points

- The causal relationship between gut microbiota and CAD development has yet to be confirmed.
- It is imperative to understand the potential direct and indirect causal roots between gut microbiota and CAD development via microbial metabolites and interaction with the immune system.
- Dynamic elements including our diet and demographic factors such as age, sex, and ethnicity can also affect our gut microbiota and CAD development and complicate this matter.
- Interdisciplinary approaches are required to shed light on the factors involved in the modulation of gut microbiota and its association with CAD development.
- Elucidating the system-level multifaceted web of factors involved in microbiome-mediated mechanisms and human health and disease can guide novel preventative and therapeutic interventions for CAD.

## Introduction

High serum cholesterol (hypercholesterolemia) is a well-documented risk factor for the most prevalent form of cardiovascular disease (CVD) known as coronary artery disease (CAD) [1–3], which is one of the leading causes of morbidity and mortality worldwide [4, 5]. Other established risk factors for CVD include hypertension, diabetes mellitus, obesity, and a sedentary lifestyle [6]. The buildup of cholesterol-containing deposits (plaque) inside the artery walls can lead to atherosclerosis [7], which is expected to cause 12 million coronary deaths annually by 2030 [8]. Hypercholesterolemia can have a genetic origin [9, 10] and affect bodily functions that are mainly responsible for cholesterol homeostasis in the body, including de novo synthesis, catabolism in the liver and secretion into bile, and intestinal absorption [11].

Cholesterol in the body originates from two sources and is synthesized de novo in the liver or can enter our body via our diet and cholesterol-rich foods. About one fourth of the cholesterol in the body comes from dietary intake (exogenous) and the rest is synthesized de novo (endogenous) via the mevalonate pathway [12, 13]. The cholesterol synthesized within the body is classified as either high-density lipoproteins (HDL) cholesterol or low-density lipoproteins (LDL) cholesterol, the latter of which can enter the circulatory system and becomes a key marker of CAD [14]. By contrast, HDL cholesterol is inversely associated with CAD [15] and has anti-atherogenic functions by exerting anti-inflammatory and anti-oxidative effects and promoting reverse cholesterol transport (RCT), which can eliminate LDL cholesterol [16]. However, HDL may lose its anti-atherogenic properties and becomes pro-atherogenic (dysfunctional) under conditions such as inflammation, diabetes, and oxidative stress [16]. Moreover, elevated LDL cholesterol is a risk factor for CAD [17], which may be due to the uptake of LDL cholesterol particles by macrophages that leads to foam cells and atherosclerosis [18].

**Fig. 1:**
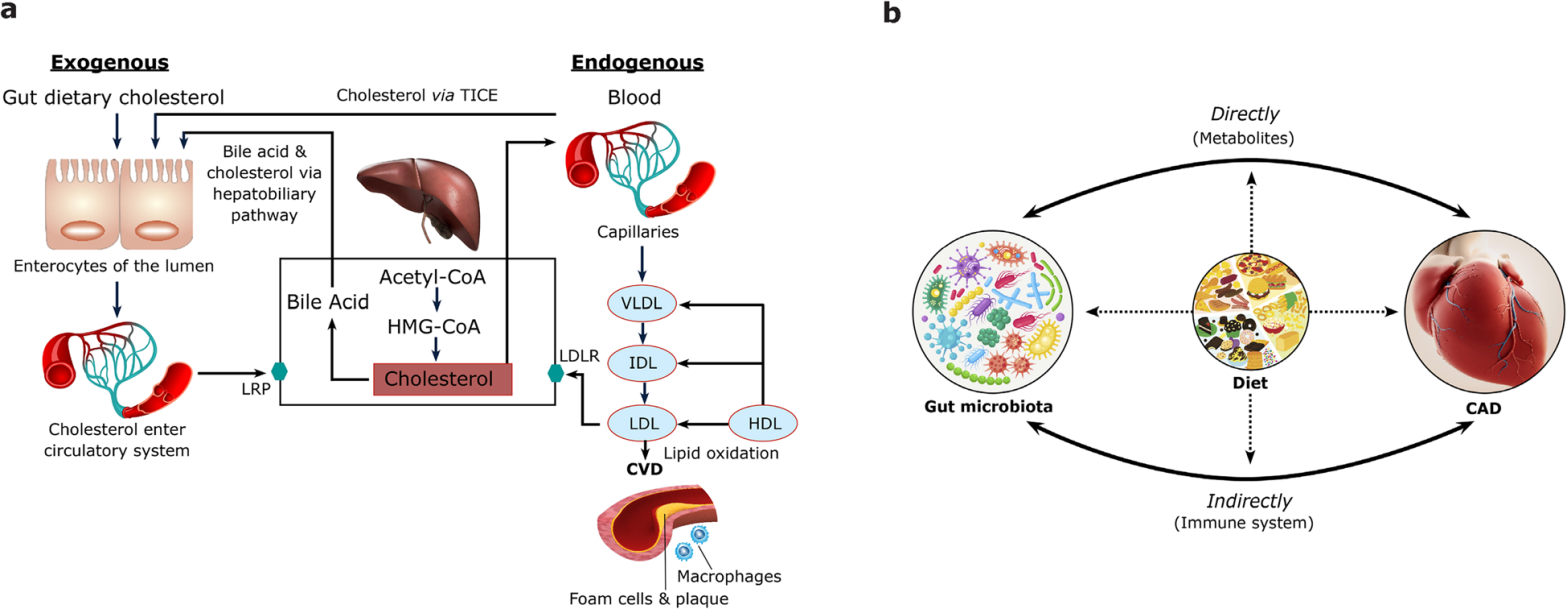
Cholesterol, gut microbiota, and CAD. **a** Exogenous and endogenous sources of luminal cholesterol. **b** The multifaceted mechanisms involved in CAD development. The gut microbiota can directly (via metabolites) and indirectly (via the immune system) lead to CAD

The gut lumen plays an eminent role in controlling the body’s cholesterol balance and is responsible for exogenous intake via cholesterol absorption [19]. Luminal cholesterol can come from different sources and is mainly derived from (i) our diet, (ii) bile via the hepatobiliary pathway [20], and (iii) de novo cholesterol via the transintestinal cholesterol efflux (TICE) pathway [21, 22] (Fig. 1a). In the liver, cholesterol is metabolized into bile acid and is secreted into bile via the hepatobiliary pathway where the ATP-binding cassette transporter, G5/ATP-binding-cassette transporter G8 (ABCG5/G8), plays a key role in cholesterol efflux from hepatocytes into bile [23]. TICE is an alternative route to the hepatobiliary pathway, where cholesterol from the blood can directly enter enterocytes through LDL receptors (LDL-R) and is effluxed by ABCG5/G8 and the ATP-binding cassette transporter B1 (ABCB1a/b) into the lumen [22]. The cholesterol content of the lumen is then either absorbed into enterocytes via Niemann-Pick C1-like 1 (NPC1L1) and incorporated into chylomicrons for entry into the circulatory system [19], or is reduced by gut microbiota to poorly absorbable coprostanol (5B-Cholestan-3B-ol) [24–26], which is mostly excreted.

Aside from the complex interplay of numerous cholesterol sources in the body, many other factors can affect cholesterol balance and CAD development including our gut microbiota. To date, associations between an altered gut microbiome composition and metabolic disorders such as obesity, diabetes mellitus, and CVD (independent of age, sex, and host genetics) [27, 28], including atherosclerosis, dyslipidemia, hypertension, and heart failure have been suggested [29–31]. Such links can be through direct (via metabolites) and indirect pathways (via the immune system) [27, 32]. The adult human gastrointestinal tract harbors 100 trillion bacteria belonging to at least several hundred species [33]. The gut microbiota plays multiple critical roles in the maintenance of their host health, including helping host nutrition and energy harvest, intestinal epithelial homeostasi s[34, 35], drug metabolism and toxicity [36], immune system response [37], and protection from pathogens [38]. These microorganisms can also generate microbial products such as uremic toxins [39], bile acids [40], trimethylamine-n-oxide (TMAO) [41], short chain fatty acids (SCFA) [42], lipopolysaccharides (LPS) [43], nitric oxide [44], vitamin K [45], vitamin B complex [46], gut hormones [47], and neurotransmitters [48], which can alter host metabolism and affect bodily functions in health and disease states. Susceptibility to atherosclerosis, for example, has been demonstrated to be transferable by microbiota transplantation in murine models [49]. To date, many infectious agents have been linked to atherosclerosis including *Helicobacter pylori*, *Cytomegalovirus*, Hepatitis C virus, *Chlamydia pneumoniae*, and *Porphyromonas gingivalis* [50]. Interestingly, a study by Mitra et al. showed that microbiota displayed differences between symptomatic and asymptomatic atherosclerotic plaques, with asymptomatic plaques having an increased abundance of host microbiome associated families including *Porphyromonadaceae*, *Bacteroidaceae*, *Micrococcacaea*, and *Streptococcacaea* [51]. In contrast, symptomatic atherosclerotic plaques contained an increased abundance of pathogenic microbiome families including *Helicobacteracaea*, *Neisseriaceae*, and *Thiotrichacaea* [51]. Moreover, gut microbiota dysbiosis as a result of the disruption to the overall state of gut microbiota has been associated with increased inflammation, which is linked with the development of atherosclerosis [52]. Recently, alterations in the gut microbiota and its metabolites have also been associated with hypertension and vascular dysfunction [53, 54]. Heart failure has also been associated with specific gut microbial species such as increased *Escherichia coli, Klebsiella penumoniae*, and *Streptococcus viridans* [55]. One study has shown that patients with symptomatic stroke and transient ischemic attack have an altered gut microbiota with increased opportunistic pathogens including *Enterobacter*, *Megasphaera*, *Oscillibacter*, and *Desulfovibrio* [56]. Furthermore, the gut microbiota have the capacity to contribute to substantial variation in blood lipid composition [57], which can affect CAD development. For example, Firmicutes such as *Lactobacillus reuteri* are associated with higher HDL [58], whereas the genus *Eggerthella* is associated with decreased HDL cholesterol [57].

Currently, the causal relationship between the gut microbiome and CAD development remains unclear since many other demographic factors such as age, sex, and ethnicity can not only affect gut microbiota and cholesterol levels but also our diet, which is another component affecting our gut microbiota and whole body cholesterol levels. Thus, cholesterol regulation in the body is a complex mechanism with factors that are intertwined in a multifaceted system (Fig. 1b). Therefore, further studies are needed to understand the underlying mechanisms and identify which microbial strains or their metabolites are responsible for CAD development. This review will discuss the dynamic elements involved with the gut microbiota and their effects on hypercholesterolemia and CAD development via direct and indirect pathways. In addition, we will address the current challenges to prove causality, discuss the gaps in knowledge, and propose the potential role of nanotechnology in uncovering the underlying mechanisms involved in CAD development and as well as a microbiome-targeted therapeutic tool.

## Effects of gut microbiota on CAD

### Direct effect

Gut microbiota can directly affect hypercholesterolemia and CAD development via metabolite production such as bile acids, coprostanol, short chain fatty acids, and trimethylamine-n-oxide production.

#### Bile acid modulation

**Fig. 2:**
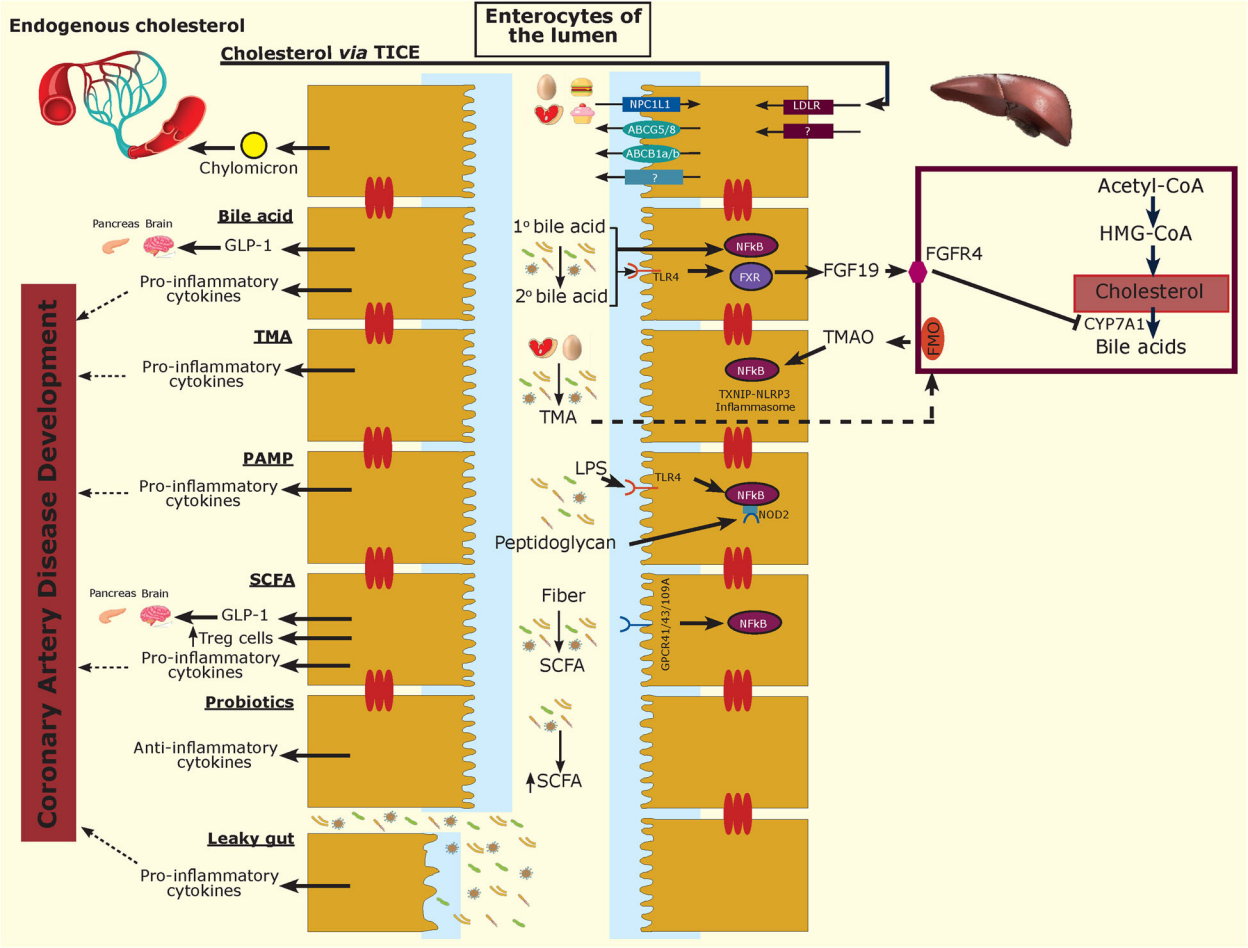
Multifaceted mechanisms affecting CAD. Exogenous and endogenous sources of luminal cholesterol and diet, and the gut microbiota mechanisms involved in affecting the immune system and CAD development

The gut microbiota can affect the regulation of cholesterol metabolism in the liver [40, 59] and play a role in altering bile acids that can influence systemic cholesterol levels [60] (Fig. 2). Bile acids, formed by the rate-limiting enzyme cholesterol 7-alpha-hydroxylase (CYP7A1) [61], are the main metabolites of cholesterol in the liver that help in the absorption of fats, nutrients, and lipophilic vitamins [62] and also the regulation of lipids, glucose, and energy metabolism [63, 64]. Primary bile acids are conjugated to amino acids taurine or glycine to form bile salts that are secreted into bile and stored in the gallbladder until they are released into the small intestine where they emulsify fats and forms micelles which are absorbed into enterocytes [62]. In the gut, the primary bile acids such as cholic acid (CA) and chenodeoxycholic acid (CDCA) become deconjugated by the gut microbiota and bile salt hydrolase (BSH) to form secondary bile acids, including deoxycholic acid (DCA), lithocholic acid (LCA), and ursodeoxycholic acid (UDCA) [62, 65]. All conjugated and unconjugated bile acids in the lumen can be reabsorbed (95%) and transported back to the liver, except for UDCA and LCA, which are mostly excreted in feces [61]. Signaling molecules such as bile acids in the gut can also activate nuclear receptor farnesoid X receptor (FXR) and the membrane G protein-coupled bile acid receptor Gpbar-1 (aka TGR5) [62]. Through this mechanism, bile acids can downregulate bile acid synthesis [66], which can lead to increased cholesterol levels. The order in which bile acids can activate FXR are CDCA>DCA>LCA>CA [67]. FXR can induce fibroblast growth factor 19 (FGF19), which activates fibroblast growth factor receptor 4 (FGFR4) and suppresses CYP7A1 to downregulate bile acid synthesis [68]. FXR can also reduce bile acid uptake into hepatocytes and increase biliary secretion of bile acid by increasing the expression of ATP-binding cassette subfamily B member 11 (ABCB11) [66, 69]. Primary and secondary bile acid ratios may be implicated in hypercholesterolemia and CAD development. For example, in a study by Myerhofer et al. [70], the plasma primary bile acids were reduced, and the ratio of secondary to primary bile acids was higher in heart failure patients [70]. Bile acids can also play a role in cardiovascular function by reducing heart rate through regulating channel conductance and calcium dynamics in sin-atrial and ventricular cardiomyocytes and regulating vascular tone [70]. In addition, we propose that the gut microbiota modulating bile acid ratios, if unbalanced and in an unhealthy state, could lead to reduced secondary bile acids, which can increase primary bile acids such as CDCA, activate FXR, downregulate bile acid production, and thus increase cholesterol and CAD development. Thus, the gut microbiota and the underlying mechanisms involved need to be further investigated.

#### Coprostanol production

Certain gut microbiota have long been known to possess the ability to convert absorbable cholesterol to coprostanol, a reduced non-absorbable sterol, which is excreted in feces [71–73]. Coprostanol production in humans starts during the second half of the first year of life [26] and is sex-dependent, with young women being high converters compared to young males [74]. Furthermore, currently, the rate of microbial cholesterol-to-coprostanol conversion in human populations is believed to be bimodal, with high converters showing almost complete cholesterol conversion and low converters with coprostanol representing less than one third of the fecal neutral sterols content [75, 76]. To date, isolated cholesterol-reducing strains have been limited to the genera of *Eubacterium* (*E. coprostanoligenes*) and *Bacteroides (Bacteroides* sp. strain D8) [77, 78], but many remain to be uncovered. Using animal models, the oral administration of *E. coprostanoligenes* resulted in a significant decrease of plasma cholesterol concentration in dietary-induced hypercholesterolemic rabbits that lasted for at least 34 days after the last bacterial feeding [79]. For human models, there have been many studies on cholesterol metabolism in the gut [25, 26, 75, 77, 80], and an inverse relationship between the human serum cholesterol and coprostanol/cholesterol ratio in the human feces has been suggested [77, 81, 82]. However, these studies employed very small sample sizes with a limited variation of sample populations lacking diverse demographic backgrounds and included unsuccessful attempts to isolate specific microbial strains responsible for the coprostanol/cholesterol conversion. In addition, the genes or enzymes involved in the cholesterol conversion to coprostanol in the gut remain unknown [83].

#### Short chain fatty acid production

SCFAs are a microbial-derived metabolite that are formed due to the fermentation of complex carbohydrates [42, 84] (Fig. 2) affecting a range of host processes such as host-microbe signalling, energy utilization, and control of colonic pH with consequent effects on the microbiota composition and gut motility [85]. The most abundant SCFAs are acetate, propionate, and butyrate [84]. Bacteroidetes phylum members can yield acetate and butyrate, whereas Firmicutes phylum can lead to butyrate [86]. SCFAs are also positively correlated with *Alistipes putredinis*, *Bacteroides* spp., *Roseburia*, *Eubacterium rectale*, and *Faecal prausnitzii* [87]. Furthermore, they play an integral part in maintaining the intestinal barrier integrity by regulating the expression of tight junction proteins [88]. SCFAs can also lower serum lipid levels by blocking cholesterol synthesis and/or redirect them to the liver [89]; therefore, they have been suggested as a protective element in CAD development. SCFA-producing bacteria have also been reduced in certain CAD cases [29, 30] as well as in gut dysbiosis of patients with hypertension [30, 90] via activation of G protein-coupled receptors 41 (GPR41) [91]. Thus, their role in the body and their targets require further investigation.

#### Trimethylamine-n-oxide production

Dietary choline, betaine, phosphatidylcholine, lecithin, and l-carnitine [92–94] are involved in the production of TMAO, a risk factor for CAD development [40, 93] (Fig. 2) that come from many sources, including red meat, egg, fish, brassica vegetables, peanuts, and soybean [95]. Specifically, increased TMAO levels have been associated with an increased risk of death and non-fatal myocardial infarction or stroke [96]. The gut microbiota also play a role in TMAO production via (a) choline production and (b) the intermediate molecule trimethylamine (TMA) production. Only recently has the gut microbiota’s ability to produce choline via phospholipase D (PLD) enzyme been found [97]. The microbiome-derived TMA molecule can pass into host circulation and travel to hepatocytes, where it is metabolized to TMAO [94] by flavin containing monooxygenase (FMO) enzyme encoded by the *FMO* gene in the liver, kidney, and other tissues [98]. High TMAO production can consequently affect lipids [41] and lead to a 43% higher CAD risk due to the reduction of RCT and alteration in bile acid transport, composition, and pool size [92, 93, 99]. TMAO is also associated with C-reactive protein (CRP) and endothelial dysfunction in the setting of increased gut permeability and is related to increased serum levels of LPS endotoxin [100]. In addition, it can also lead to calcium release and platelet hyperreactivity [101], which can affect CAD development.

The gut microbiota can heavily influence TMAO production. Healthy individuals have high TMAO producing microbes and a ratio of 2:1 for Firmicutes to Bacteroidetes [102]. TMA production has been found in 102 genomes covering 36 species, and TMA producers include Firmicutes, Proteobacteria, Actinobacteria, and absent in Bacteroidetes [95]. Firmicutes including *Anaerococcus*, *Clostridium*, *Desulfitobacterium*, *Enterococcus*, *Streptococcus*, and Proteobacteria including *Dseulfovibrio*, *Enterobacter*, *Escherichia*, *Klebsiella*, *Proteus*, *Pseudomonas*, *Actinobacter*, *and Citrobacter* have been associated with TMA production [100]. One study found that 8 species from Firmicutes and Proteobacteria consumed > 60% of choline for TMA production: *Anaerococcus hydrogenalis*, *Clostridium asparagiforme*, *C. hathawayi*, *C. sporogenes*, *Escherichia fergusonii, Proteus penneri*, *Providencia rettgeri*, and *Edwardsiella tarda* [103]. Other gut microbiota associated with higher TMAO production include *Akkermansia*, *Sporobacter*, *Prevotella* [95], and *Ruminococcus gnavus* [104], which are associated with atherosclerotic CAD. Thus, metabolites including choline, TMA, and betaine can aid in predicting CAD development. For example, probiotics or pharmacological intervention can be utilized in order to inhibit or block specific microbial metabolic pathways to reduce TMAO producing microbes [105].

### Indirect effect

Gut microbiota can also lead to CAD development via an indirect pathway such as the manipulation of our immune system (Fig. 2). Atherosclerosis is a chronic inflammatory disease [7] triggered by atherothrombosis in which (a) superficial erosion may lead to clot formation [106] or (b) rupturing of plaques damaged by cytokines, which can lead to exposed coagulation systems resulting in inhibited blood flow and inducing CAD [107]. Thus, macrophages and innate immunity triggered by inflammation are implicated in CAD [108]. For example, a high white blood cell (WBC) count has recently been deemed a risk factor for CAD development [109]. In addition, a study by Wang et al. identified the IL-22 pathway as a novel target for therapeutic intervention in metabolic diseases, since IL-22 can improve insulin sensitivity, preserve gut mucosal barrier and endocrine functions, decrease endotoxaemia and chronic inflammation, and regulate lipid metabolism in liver and adipose tissues [110–112]. In our body, oxidized LDL (oxLDL) can also exert pro-atherogenic and pro-inflammatory effects by activating endothelial cells, macrophages, and T cells [109, 113]. Macrophages can engulf oxLDL and lead to inflammatory cytokines such as tumor necrosis factor alpha (TNF-α), interleukin 1 beta (IL-1β), IL-6, IL-18, IL-37, and foam cells, which can exacerbate CAD [109, 113, 114]. TNF-α has also been implicated in risk factors of CAD including diabetes by activating protein kinase C (PKC), which can increase the phosphorylation of insulin receptor substrates resulting in their inactivation [115]. T cells can also lead to pro-inflammatory cytokines IL-2, IL-12, and interferon gamma (IFN-**γ**) [116], which are associated with arterial stiffness [117]. Together, foam cells, T cells, and macrophages can lead to fatty streaks and consequently contribute to CAD development [19].

The community structure of our gut microbiota can greatly influence our immune system. For example, a low gene count (LGC) of gut microbiota has been correlated with high WBC counts [118], which as previously stated is a risk factor for CAD. Among our gut microbiota, the presence of *Lactobacillus reuteri* has been specifically associated with high WBC count [119]. Individuals with LGC suffer from metabolic disturbances resulting in dyslipoproteinemia and pro-inflammatory status, which can lead to CAD [120]. An LGC is also associated with a high CRP level [118] with low *Oscillibacter*, *Faecalibacterium*, and *Ruminococcus* correlating with high CRP levels [121, 122]. The expression of pattern recognition receptors (PRRs) like TLRs in the intestine is also modulated by gut bacteria that help the host navigate between pathogens through pathogen-associated molecular patterns (PAMPs) and commensal bacteria, as well as the activation of immune sensory cells [123, 124]. Furthermore, our microflora can affect regulatory T (Treg) cells, and their reduction can exacerbate infection outcomes [125] and heighten the risk of autoimmune diseases [126], allergies [127], and cancers [128]. *Prevotella*, for example, can mediate inflammatory response via toll-like receptor 2 (TLR2) activation, which can lead to inflammation and T-helper cell 17 (Th17) immune response [120]. The disease progression of myocarditis (an inflammatory heart disease) into lethal cardiomyopathy can depend on cardiac myosin specific Th17 cells imprinted in the intestine by b-galactosidase mimic peptides in commensal *Bacteroides thetaiotaomicron* and *B. faecis*, which can promote inflammatory cardiomyopathy [129]. *Clostridium* cluster IV enhances Treg cell abundance and leads to the production of anti-inflammatory molecules [130]. Thus, TLR2 is implicated in CAD pathogenesis [131]. NOD/CARD, another class of PRRs, can recognize stress responses and activate inflammation caspase by activation of inflammatory cytokines and/or activating immune system transcription factor NF-κB to result in the production of inflammatory molecules [123]. A leaky gut can also result in the translocation of gut microbiota-derived components such as PAMPs, including LPS [43], which can lead to the production of pro-inflammatory cytokines [132]. Thus, assessment of the gut microbiota can function as a potential diagnostic marker so that a pro-inflammatory state can be detected early to predict the risk of CAD development.

Gut microbiome metabolites such as SCFA can also affect the immune system, exerting an anti-inflammatory impact [133] through the activation of G protein-coupled receptors 41 (GPR41), 43 (GPR43), and 109A (GPR109A) [134] via induction of Treg cells controlled by the forkhead box P3 (Foxp3) promoter [135]. In addition, they can produce anti-inflammatory gut hormones such as glucagon-like peptide 1 (GLP-1) [136]. Although SCFAs have many positive effects, their production can also shift the bacterial balance and lead to inflammation through activating the toll-like receptor 4 (TLR4) [137]. Therefore, their role in the immune system needs to be further investigated. The gut microbiome-derived TMAO can also affect our immune system by activating TXNIP-NLRP3 inflammasomes [138], leading to the expression of inflammatory markers such as TNF-α, IL-6 [100, 139], IL-18, and IL-1B [138] that can boost plaque development in arteries by generating cholesterol-packed foamy macrophages, ultimately resulting in CAD [140] (Fig. 2). TMAO can also boost PKC/NF-κB activation, elevating the expression of vascular cell adhesion molecule 1 (VCAM-1) and monocyte adhesion [141]. Aside from influencing HDL cholesterol and anti-inflammatory properties [16], the gut microbiota and their associated metabolites can also affect the immune system through a non-inflammatory induced pathway. Primary (deconjugated by gut microbiota) and secondary bile acids, for example, can inhibit NF-κb-dependent transcription of pro-inflammatory cytokines via FXR and TGR5 receptors [120]. The activation of TGR5 can also protect against LPS-induced inflammation [142] and atherosclerosis [143]. In addition, certain cytokines such as IL-10 can have a positive effect such as by decreasing serum cholesterol and atherosclerotic plaques in mice [144] through the increased uptake and efflux of acetylated and oxLDL from atherosclerotic lesions via the induction of RCT [145]. This cytokine can also lower total cholesterol by enhancing liver resident Kupffer cells’ phagocytosis. These cells represent 80–90% of macrophages in the body [146] and may be novel targets for therapeutics. Dissecting complex interactions between immune and metabolic systems will provide insights into the biology underlying CAD and how current and future therapies might influence metabolism.

#### Diet affecting the whole system

**Fig. 3:**
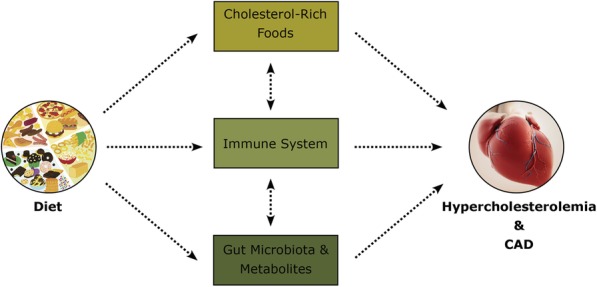
Microbiota, diet, and CAD. Diet directly and indirectly affects cholesterol levels and CAD development via the consumption of cholesterol-rich foods, can affect on the immune system, and lead to the modulation of gut microbiota and their metabolites such as bile acids, coprostanol, SCFA, and TMAO

As previously discussed, one fourth of our bodily cholesterol comes from dietary intake [12, 13]. This has led to a growing debate on whether dietary cholesterol can affect CAD development. Our diet can complicate matters by affecting cholesterol modulation and CAD development directly via consuming cholesterol-rich foods and indirectly via modifying the gut microbiota and their community structure, bile acid production, coprostanol production, SCFA production, and TMAO production. For example, beneficial modifications of gut microbiota caused by the Mediterranean diet have been shown to ameliorate obesity, inflammation, CAD, and other related metabolic alterations [147, 148]. This diet puts greater emphasis on fruits, vegetables, and legumes and has been associated with increased SCFA levels [149]. In addition, diet can affect the immune system by shifting inflammatory responses that are linked with cholesterol modulation and CAD development (Fig. 3). A study by Wilck et al. showed that high salt intake affects the gut microbiome, particularly by depleting *Lactobacillus murinus* and increasing Th17 cells and hypertension [150]. Supplementation with *L. murinus* blunted Th17 activation and ameliorated hypertension [150]. In addition, Westernized diet composed of high unsaturated fat can lead to increased Bacteroidetes and decreased Firmicutes and *Bilophila wadsworthia* (sulfite reducing microorganisms), compared to a diet composed of high saturated fat that can lead to increased LDL cholesterol [151] and *B. wadsworthia*, which is associated with dyslipidemia and increased inflammation [87, 152, 153]. High protein and high fat diets have also been associated with increased *Ruminococcus* [154] and decreased Bacteroidetes, *Clostridium coccoides*, *Bifidiobacterium*, *E. rectale*, *Akkermansia municiphila* [155–157], and increased bile acid concentration in feces, including DCA concentrations, which can cause liver cancer [155]. In addition, these diets can activate TLR4 that are associated with inflammatory responses such as pro-inflammatory cytokines, Th1, CD4, and T cells, leading to the downregulation of Treg cells [158, 159]. During a high fat diet-induced diabetes, bacteria from the intestine are translocated towards tissues and the blood, which depends on CD14 and NOD1 [160]. However, this bacteremia can be reversed via a probiotic (*Bifidobacterium animalis*), which can reduce the adherence and translocation of bacteria as well as adipose tissue and inflammation occurring during diabetes [160]. In another study, probiotic administration of *Lactobacillus casei* reduced bacterial translocation and altered the gut microbiota by increasing *Clostridium coccoides*, *C. leptum*, and total *Lactobacillus* [161]. TMAO and SCFA production can also vary, with omnivores producing more TMAO compared to vegans [92], and high fiber diets leading to higher SCFAs [152, 162] and increased gut bacterial diversity [162]. The notion of diet influencing cholesterol in the body is a continuing debate that requires further research. Although many studies have indicated a direct relationship between high dietary cholesterol and CAD, other studies suggest that the clinical effect of cholesterol in diet may be minor or negligible in disease development [151, 163–165]. This debate is likely due to our lack of understanding of the bodily system mechanisms involved in managing cholesterol levels and as well as the normal gut microbiota that vary among individuals and based on demographic and environmental factors.

Our diet can also have anti-inflammatory effects through omega-2 (n-3) polyunsaturated fats that interact with the transcription factor NF-κB and PPAR-Y, downregulate pro-inflammatory genes, and inhibit TLR4 activation, creating an anti-inflammatory response [166, 167]. Anthocyanin in our diet (e.g., blueberries), for example, is an antioxidant that can affect the gut microbiota by increasing their diversity, which may reduce inflammatory responses [168]. Pre- and pro-biotics have also been thoroughly investigated and shown to improve the gut environment by intestinal barrier enhancement, regulation of immune functions, and the prevention of pathogenic infections [169]. They have been associated with decreased inflammation [170] and increased SCFA, Bacteroidetes, *Bifidiobacterium*, and decreased Firmicutes [171]. Orally administered probiotics can even reduce cholesterol by 22–33% due to BSH activity [172]. For example, probiotics *Lactobacilli* and *Bifidobacteria* can deconjugate bile acid and increase excretion by (a) increasing demand of cholesterol for de novo synthesis of bile acid or by (b) reducing cholesterol solubility and decreasing its absorption [65]. Although pre- and probiotic usage is increasing in popularity, questions remain with respect to specific immune and physiological effects they may have on health and disease and thus further studies are needed.

#### Microbiota, demographic factors, and CAD

In the era of precision medicine, a key challenge is to bridge the gap in our knowledge about interactions among demographic factors, the gut microbial composition, and the pathophysiology of the cardiovascular system [173, 174]. Beyond environmental and social differences between men and women (e.g., occupational hazards, lifestyle, social stresses, and access to healthcare) that can contribute to gender differences in disease development, sex chromosomes, and sex hormones can also contribute to sex- and gender-related differences in CAD [173, 175]. More specifically, sex differences in lipid and lipoprotein metabolism have been shown recently [176, 177], as well as sex-specific considerations for the treatment of dyslipidemia [176]. Although CAD is considered a “men’s disease,” a growing body of evidence is also revealing the importance of CADs in women and increasing the awareness of sex- and gender-related differences in the occurrence, diagnosis, management, and outcomes of CADs [178, 179]. Women, for example, are more prone to this disease in later stages of their life [173]. This may be due to changes in hormones and menopause, which can affect cholesterol rates with the cessation of estrogen production, shifting lipoproteins toward LDL and away from HDL cholesterol in women [180]. Sex-differences are also associated with the overall gut microbiota structure [181, 182], which as previously discussed is associated with CAD development. For example, in a study by Takagi et al., significant increases in genera *Prevotella*, *Megamonas*, *Fusobacterium*, *Megasphaera*, *Bifidiobacterium*, *Ruminococcus*, and *Akkermansia* were found in males and females, respectively [182]. However, males and females did not differ significantly in their microbial diversity [182]. Studies based on sex- and gender-related differences in gut microbial composition and CAD development are still rare and need to be expanded in number and depth [178].

Ethnicity differences, though often overlooked in studies, are known to affect hypercholesterolemia and CAD development. Ethnicity differences can capture biological variations derived from social, economic, and cultural differences, human genetic variation, and biogeographical ancestry divergences, as well as lifestyle and dietary differences [183]. Risk factors of CAD development including smoking, blood pressure, obesity, and cholesterol can also vary among different ethnicity groups [184, 185], resulting in certain groups having an earlier onset and worse outcomes of CAD. For example, South Asians are a high-risk ethnic group and have lower rates of physical activity [186]. African Americans residing in the USA also have a higher risk for CAD development, which may be due to lifestyle, environmental factors, and socioeconomic factors such as lower education, higher poverty, higher uninsured rates, and decreased access to healthcare [187, 188]. In addition, African Americans also have a diet with relatively higher sugar, higher sodium, and lower potassium [187] contents that can lead to higher blood pressure. In addition, ethnicity and dietary differences are associated with variations in microbial composition and abundance [181, 189, 190] and even more strongly with gut microbiota than other factors such as genetics [191], age, sex, and BMI [183]. For example, comparative studies of the microbiome in rural and urban areas in healthy individuals have reported that populations residing in non-Western and/or rural areas have a higher bacterial diversity when compared with populations in America and Europe [162, 192]. In another study by Deschasaux et al., there was a higher gut microbial diversity observed within the Dutch population and the smallest diversity in South Asians, with Ghanaians, Turks, and Africans in the middle [193]. Increased Firmicutes and decreased Bacteroidetes were also observed in the Dutch population, while increased Actinobacteria was observed in the South Asian populations [193]. The interplay between demographic factors such as sex, age, and ethnicity and their links with our diet, gut microbial composition, and CAD development illustrate the complexity of our bodily factors involved in health and disease states. Therefore, greater research efforts are required to understand these factors involved in gut microbial changes and CAD development.

**Fig. 4:**
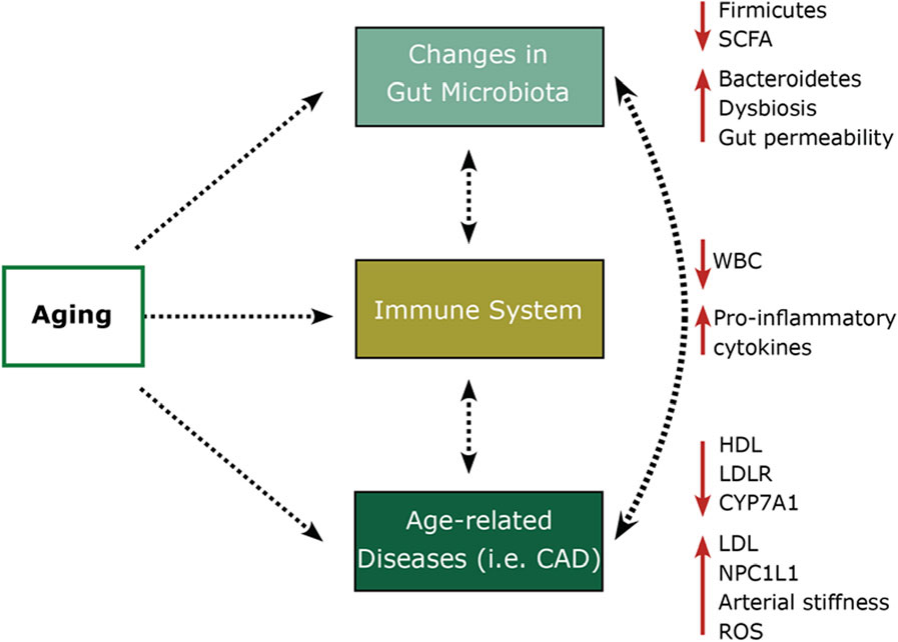
Microbiota, aging, and CAD. Selected aging-related mechanisms involved in systemic inflammation and adverse health outcomes. SCFA short chain fatty acid, WBC white blood cells, HDL high-density lipoprotein, LDLR low-density lipoprotein receptor, CYP7A1 cholesterol 7-alpha-hydroxylase1, LDL low-density lipoprotein, NPC1L1 Niemann-Pick C1-like1, ROS reactive oxygen species

Cholesterol in the body can also be affected by the natural aging process, which is an uncontrollable risk factor that can lead to the dysregulation of whole-body cholesterol metabolism (Fig. 4) [194]. By 2030, 1 billion individuals are projected to be over 65 years old [195]. Generally, the aging process is associated with progressive deterioration in the structure and function of the heart, as well as the vasculature that can contribute to CAD development [196]. In addition, through the aging process, LDL cholesterol levels can increase, and HDL cholesterol levels can decrease [197], which can lead to increased rates of CAD development. Other factors caused by the aging process include decreasing CYP7A1 enzyme activity (decrease regulation of bile acid synthesis), decreasing hepatic LDL cholesterol receptors (decrease LDL cholesterol clearance), and increasing NPC1L1 (mediator of cholesterol absorption) [198, 199]. Aging also affects the gut microbial community due to the accumulation of disorders, changes in diet, a decrease in exercise and mobility, and the use of certain medications [121, 200]. However, contradictory findings have also been found suggesting no significant differences in the gut microbial structure of participants from various age groups [182, 201]. Overall, it is safe to conclude that aging is associated with increased gut dysbiosis and is inversely associated with gut microbial diversity [202]. In addition, the abundance of genes involved in SCFA production also decreases with age [203]. Aging affects the immune system, with systemic inflammation being one of the hallmarks of aging and one of the causes of increased risk for many age-associated diseases including CAD, diabetes, and cancers [109]. Furthermore, aging is modulated by a positive feedback loop in which chronic systemic inflammation in older people is associated with developing age-related diseases which then lead to increased inflammatory responses through these conditions as well [109]. For these reasons, the inclusion of demographic factors such as age, sex, and ethnicity is a must for studies in the era of precision.

#### Microbiota in precision medicine

Currently, many techniques can be utilized in order to parse out gut microbiome associations with human immunology [204], neurology [205], and endocrinology [206]. Due to such associations and their potential in precision medicine [207], the human microbiome is being vastly studied as a therapeutic target using fecal microbiota transplantation, probiotics, and prebiotics. Albeit, for the majority of diseases, mechanistic insights and translational applications are still scarce. The human microbiome is compositionally and spatiotemporally very fluid and intra- and inter-individual variations within the microbiome can affect drug efficacy and side effect profiles, either via direct biotransformation of drugs or indirect mechanisms such as microbial interactions with the host immune system. Herein, we discuss multiple emerging strategies for the precise manipulation of complex microbial communities to improve CVD treatment outcomes. In the future, we anticipate a positive shift towards an inclusive view of precision medicine that encompasses both human and microbial genomes as well as their combined metabolic activities.

#### Microbiota and pharmacological therapy

Current modalities to treat hypercholesterolemia and CAD include pharmaceuticals that can effectively reduce cholesterol levels and are utilized for the treatment of hypercholesterolemia and CAD prevention. Hydroxy-methyl-glutaryl-coenzyme A (HMG-Co A) reductase inhibitors, also known as statins [208], can affect the rate-limiting enzyme in cholesterol synthesis [209] and have revolutionized the treatment of hypercholesterolemia. This class of drugs has demonstrated significant abilities to lower total cholesterol, LDL cholesterol, and triglyceride, and increase HDL cholesterol by 18%, 25%, 11%, and 5% as shown by various studies [210, 211]. Despite statin’s efficacy, their effect on non-LDL cholesterol is limited; therefore, other drugs targeting non-LDL cholesterol may complement statins in reducing cardiovascular risks [208]. Ezetimibe, for example, is another cholesterol-reducing drug that reduces LDL cholesterol by decreasing intestinal absorption of dietary and biliary cholesterol via blocking NPC1L1 [212]. In one randomized controlled human trial, ezetimibe (10 mg/day) reduced cholesterol absorption by 54% compared with placebo and reduced total cholesterol and LDL cholesterol by 15% and 20%, respectively [213]. Although many pharmacological agents are available to reduce cholesterol, they are often suboptimal, expensive, and accompanied by many unwanted side effects [214]. Statins, for example, are associated with skeletal muscle, metabolic and neurological effects, and other possible side effects [215]. The cessation of statin treatment is also associated with worse cardiovascular outcomes [216]. Furthermore, ezetimibe is marked with a compensatory feedback upregulation of endogenous cholesterol synthesis in the liver [164] and can also increase TICE [217], which can lead to increased serum cholesterol. In addition, the inhibition of hepatic NPC1L1 can increase the cholesterol saturation index in bile and has the potential to lead to gallstones [218]. Therefore, although these conventional treatments have improved quality of life and outcomes for many patients, CAD and hypercholesterolemia remain a progressive disease. Another challenge is that the gut microbiota can directly and indirectly influence drug response either by interfering with drug pharmacokinetics or pharmacodynamics [219, 220]. For example, simvastatin, rosuvastatin, and atorvastatin (3 commonly prescribed statin medications) display evidence for modulation by the gut microbiome [219]. Metabolites such as bile acids can also influence drug pharmacokinetics by competing with drug transport mechanisms across the gut lumen, or by influencing uptake in the liver [219]. Further investigation of the molecular mechanisms by which the gut microbiome contributes to CVD and drug response will enable us to improve outcomes for CVD patients and move toward microbiome-informed precision medicine.

#### Microbiota and nanomedicine-based approaches

Nanomedicine is defined by the US National Institute of Health (NIH) as the application of nanotechnology in controlling biological systems, treatment, diagnosis, and monitoring of diseases [221]. This new branch of medicine is a multidisciplinary field of science focused on the development of diagnostic and therapeutic nano-objects that, at least in one dimension, lie within the range of 0.1–100 nm [222]. Nanoparticles in nanomedicine have been employed in unique medical applications, including the delivery of toxic biomolecules to targeted sites such as cancerous tissue but not healthy cells, the sensitive and precise imaging to detect disease at very early stages, and the crossing of difficult barriers (e.g*.*, the blood-brain barrier) to deliver imaging and therapeutic molecules to specific diseased/damaged tissues [223]. Studies involving the rational delivery and targeting of pharmaceutical, therapeutic, and contrast agents, as well as tissue engineering, are at the forefront of nanomedicine [224]. For instance, in the field of drug delivery, nanocarriers have shown the capability to minimize drug degradation, improve drug absorption and diffusion through the epithelium, modify pharmacokinetic profiles, and enhance intracellular penetration and distribution [225]. However, to date, fewer than expected numbers of therapeutic nano-formulations have been approved by the US Food and Drug Administration (FDA). Nevertheless, the large number of proof-of-concept studies on nanomaterials, the tremendous investment in the clinical development of nanotechnology-based platforms, and continuing efforts in design and preclinical evaluation of new nanoparticle products together with the recent efforts on debugging nanobiointerfaces [226] all suggest a flourishing future for the field of nanomedicine [227], with numerous applications and enormous potential in each.

**Fig. 5:**
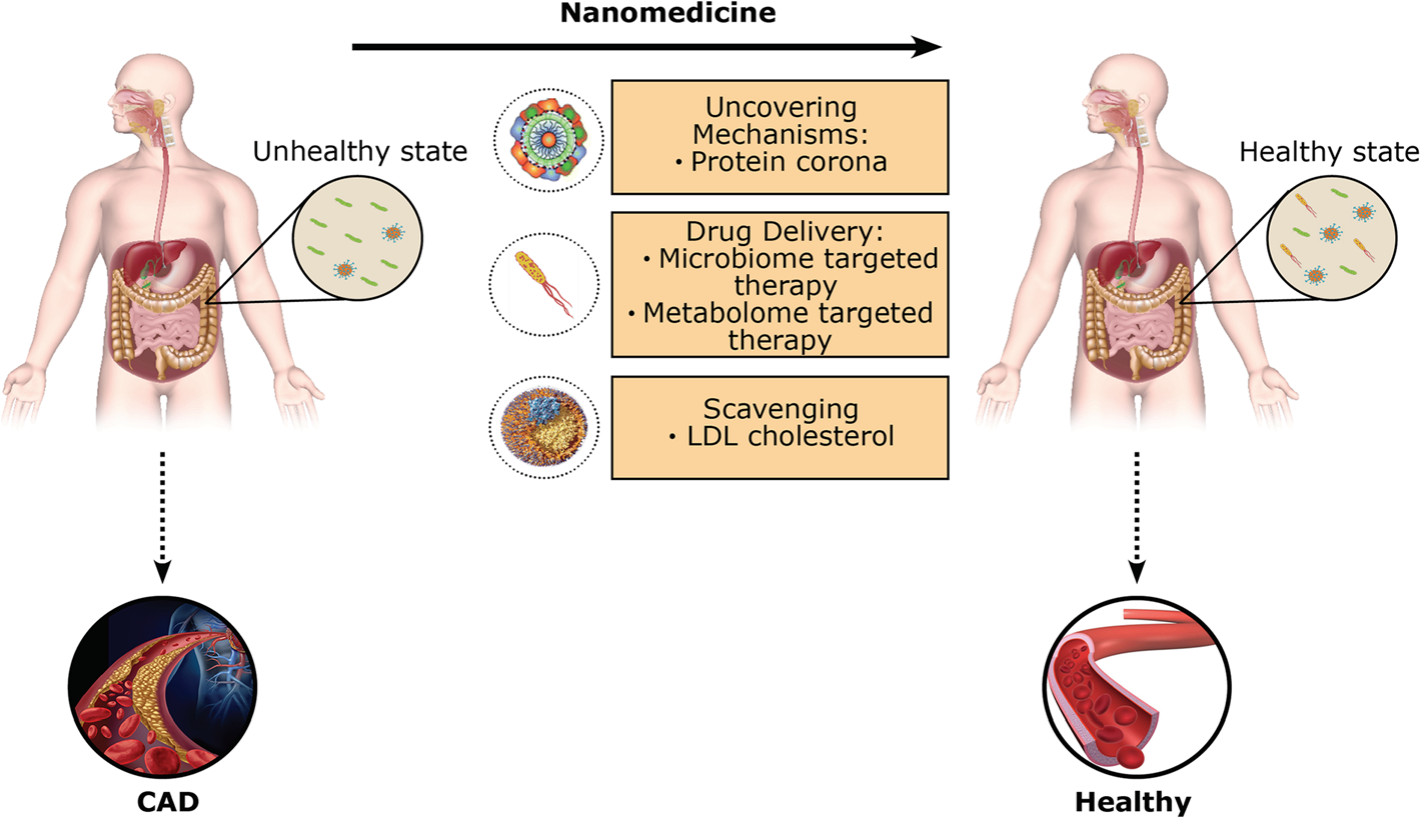
Nanomedicine, microbiota, and CAD. Nanoparticles in nanomedicine have many applications that can aid in the prevention, diagnosis, and treatment of CAD. The utilization of nanoparticles to understand the underlying bodily mechanisms (i.e., protein corona analysis), drug delivery (i.e., microbiome- and metabolome-targeted therapies), and scavenging particles (i.e., for LDL cholesterol modulating the immune system) can lead to a healthier gut microbiome and immune system that result in better overall healthy state clear of CAD development

Further developments in nanomedicine may also provide solutions to many unresolved problems in modern medicine including hypercholesterolemia and CAD (Fig. 5). The study of the relationship between gut microbiota and disease pathogenesis has proven a difficult task, particularly in teasing out causation. Nanoparticles in nanomedicine can help us understand the underlying mechanisms involved in CAD development. One useful aspect of in vivo application of nanoparticles is the formation of the biomolecular/protein corona (i.e., a layer of biomolecules which covers the surface of nanoparticles upon their interactions with biological fluids [228–230]). In 2014, we found that the protein corona profiles of patients with different diseases were substantially different despite the conventional plasma analysis showing negligible variations [231]. This effect is referred to as the “disease-specific protein corona” [232], which has been replicated elsewhere [233–235] and used for early detection of diseases including neurodegenerative diseases [236]. We recently revealed that the sensitivity, specificity, and prediction accuracy of disease detection of protein corona are enhanced by using nanoparticles with different physicochemical properties (i.e., called a protein corona sensor array technology) [237]. Another potential approach to better analyze plasma proteins and get useful information regarding CAD development could be magnetic levitation (MagLev). We have recently levitated plasma proteins using superparamagnetic iron oxide nanoparticles and revealed that the levitated plasma proteins create ellipsoidal patterns [238]. Using machine learning and liquid chromatography mass spectroscopy approaches, we then demonstrated that the patterns of the levitated plasma proteins contain useful information regarding the health spectrum of plasma donors [239]. This strategy can be very helpful and feasible for monitoring the interactions between gut microbiota patterns and CAD. Using advanced data analysis, one can define the protein/biomolecular patterns with strong associations to the variations of gut microbiota profiles and the occurrence and/or progression of CAD [240]. The knowledge about the role of important biomolecular variations may provide a valuable opportunity not only for the early detection of CAD based on the specific gut microbiota patterns (which in turn affect plasma biomolecules’ compositions) but also for developing novel therapeutic approaches based on the manipulation of gut microbiota using oral nanotechnologies.

Current prospective diagnostic and therapeutic applications include imaging, tissue engineering, the delivery of conventional drugs, proteins and genetic material, and scavenging of LDL cholesterol [241–244]. For example, heparin- and chitosan-conjugated magnetic nanoparticles have shown great potential in removing LDL cholesterol from blood plasma [245]. Nanoparticles can also modulate the immune system and have been used to induce anti-inflammatory effects [246, 247]. Broad-spectrum ROS scavenging nanoparticles, for example, have been utilized in mice studies to effectively decrease oxidative stress and local and systemic inflammation [248]. Furthermore, chitosan nanoparticles induce anti-inflammatory effects by decreasing the permeability of intestinal epithelial monolayer and the secretion of pro-inflammatory cytokines [247]. In addition, nanoparticle-based inhibitors of TLR signaling have been used to decrease inflammation and treat inflammatory diseases [249].

Although nanomedicine has shown a considerable and growing capacity for the diagnosis and treatment of CAD [250], its application in the modulation of gut microbiota that can affect CAD development is still under investigation. Very recently, we proposed several nanotechnology-based strategies to control gut microbiota composition [251]. Through modulating the gut microbiota in favor of a healthy state, we can directly (via metabolites) and indirectly (via the immune system) affect CAD development in a positive manner (Fig. 4). To that end, nanoparticles can be utilized to deliver specific gut microbiota associated with (i) increased HDL, (ii) increased SCFA, (iii) decreased LPS, and (iv) decreased pro-inflammatory cytokines. Scavenging nanoparticles can also be optimized for the uptake and removal of (i) LDL cholesterol, (ii) LPS, (iii) pro-inflammatory cytokines, and (iv) TMAO. These mechanisms have great potential to aid in the prevention, diagnosis, and treatment of CAD and can be utilized to replace current pharmaceutical agents that have various negative side effects. However, challenges in designing safe and efficient nanoparticles for the prognosis and treatment of CAD still remain. For example, targeted species may be shielded by the protein corona on the surface of nanoparticles [252], which can lead to mistargeting and reduced efficacy in the treatment of CAD. Furthermore, the protein corona can affect the drug-release profile of nanocarriers [253]. Thus, further investigation of the biological identity of these novel therapeutic platforms is required in order to diagnose and treat CAD.

#### Other challenges of clinical microbiome studies

The integration of the human gut microbiome into clinical designs and settings is not an easy task and can be faced with many challenges. Typically, the human microbiota remains stable for years [254]. Despite the long-term stability and plasticity within the gut environment, inter- and intra-variability among individuals is important to consider. Intra-variability can be due to infant transitions (i.e., birth gestational age [255], type of delivery [256], and methods of milk feeding [257]), age [201], and environmental factors such as antibiotic [258–261] usage. Furthermore, inter-variability of gut microbiota can be due to sex, enterotypes, body mass index (BMI), and external factors such as lifestyle, exercise frequency, ethnicity, dietary, and cultural habits [262, 263]. This inter and intra-variability can complicate studies that aim to identify biomarkers and investigate the gut microbiome composition and function as group comparisons. Thus, integrating microbiome science into clinical practice can be achieved by accounting for the variation within CVD patients in order to identify biomarkers and therapeutics.

Sample collection for studying the gut microbiome (i.e., stool samples) can also lead to many challenges, with no standard protocol and consensus available for quality assurance and downstream analysis. For example, the gut microbiome contains distinct microbial consortia in saliva, upper GI tract, lower GI tract, and fecal samples [264]. The upper GI has shown increased *Gemella*, *Veillonella*, *Neisseria*, *Fusobacterium*, *Streptococcus*, *Prevotella*, *Pseudomonas*, and *Actinomyces,* while the lower GI has shown increased *Faecalibacterium, Ruminococcus*, and *Bacteroides* [264], which can produce methodology challenges. In addition, the composition of faecal bacterial communities can be affected by factors including experimental design and procedures such as collection, storage, and DNA extraction [265]. It has been shown that the fecal microbiome is not a representative of the mucosal microbiome, and it is crucial to move beyond the monolithic “stool-centric” viewpoint [264]. In addition to the type of samples, longitudinal sampling can increase our understanding of the steady-state, but certainly relay a burden on the patients.

Finally, within the last decade, the surge of gut microbiome studies can be attributed to the development of cost-effective high throughput next generation sequencing (NGS) technology and “omics” data such as human genomic, metabolomic, and proteomic data [266]. NGS technology coupled with advances in bioinformatics has revolutionized the field of microbiome and supplanted culture-based approaches, permitting the analysis of increasingly complex characteristics of the microbiome; however, limitations still exist. For example, 16S rRNA sequencing can lead to a uni-kingdom outlook on bacteria, but it is vital to consider all aspects of life including fungi, protozoa, and viruses. Metagenomic studies can widen the scientific lens into a multi-kingdom view, but also contain limitations. For example, a significant proportion of the data cannot be assigned a function due to a lack of close matches in reference databases [267], specifically viral data [268]. Thus, these complex omics data require specialized statistical models to take into account factors such as compositionality, sparsity, batch effects, technical noise, sampling noise, and spatiotemporal variation. Interpreting “omics” data can also produce challenges, since changes in the abundance of specific gut microbiota may not be extrapolated to having a protective or detrimental effect on the host [269]. For example, in a study by Vandeputte et al., the absolute quantity of microbes (measured using quantitative microbiome profiling) was preferred and utilized over the classic relative abundance profiling, since the latter cannot provide information about the extent of directionality of changes in taxa abundance or metabolic potential [270, 271]. Building a knowledge base to consolidate the disconnected pieces of knowledge in the field of microbiome, as well as additional innovations including natural language processing, text mining, taxonomic representations, and field wide vocabulary standardization in microbiome research, can accelerate our understanding and aid in moving towards causality [272]. Therefore, further investigations and improvements in quality control, methodology, and pipelines used are required in order to develop global models of gut ecosystem dynamics.

## Conclusions

To fully understand the role of gut microbiota in human health and to guide therapeutic interventions for hypercholesterolemia and CAD development, it is critical that we elucidate the interconnected bodily factors that work together to affect gut microbiota and disease development. Further investigations into these complex mechanisms (e.g., through advanced nanomedicine technologies, data sciences, and incorporation of factors such as ethnicity and sex) are integral to shed light on gut bacterial-mediated mechanisms, which in turn can lead to more efficacious and high-precision microbiome-based CAD preventative and therapeutic approaches which can eventually reduce the societal and economic costs of CAD.
